# Nursing Dental Coping Belief Scale among Portuguese healthcare professionals (NDCBS-PT): validation and associated factors

**DOI:** 10.3389/fpubh.2026.1950334

**Published:** 2026-09-21

**Authors:** Joana Pombo-Lopes, Paulo Mascarenhas, Jorge Fonseca, José Grillo-Evangelista

**Affiliations:** 1 Egas Moniz Center for Interdisciplinary Research (CiiEM), Egas Moniz School of Health & Science, Campus Universitário, Almada, Portugal; 2 Aging Lab, Center for Interdisciplinary Research (CiiEM), Egas Moniz School of Health & Science, Almada, Portugal; 3 Morphology Lab, Egas Moniz Center for Interdisciplinary Research (CiiEM), Egas Moniz School of Health & Science, Almada, Portugal; 4 GENE—Artificial Nutrition Team, Department of Gastroenterology Hospital Garcia de Orta, Almada, Portugal

**Keywords:** caregiver beliefs, locus of control, nursing care, oral health, psychometrics, validation

## Abstract

**Background:**

Oral health is a critical yet frequently overlooked component of global public health, with significant implications for overall health and healthcare costs. In countries like Portugal, where public dental coverage is limited, non-dental healthcare professionals are primarily responsible for monitoring oral health. This study aimed to adapt and validate the Nursing Dental Coping Belief Scale for Portuguese healthcare professionals (NDCBS-PT) and identify factors associated with their beliefs to develop effective interventions and ensure equitable care for dependent populations.

**Methods:**

This cross-sectional psychometric study followed a five-stage international protocol. Structural validity was tested via confirmatory factor analysis (CFA) using the weighted least squares mean and variance (WLSMV) estimator. Internal consistency and temporal stability were evaluated using ordinal alpha, McDonald’s omega total (ωt), and intraclass correlation coefficients (ICC). Multivariable associations were examined using robust linear models to identify factors independently associated with NDCBS-PT scores.

**Results:**

A total of 304 healthcare professionals were included (82.6% female; mean age 44.5 years). The correlated four-factor model - internal locus of control (IL), external locus of control (EL), self-efficacy (SE) and oral healthcare beliefs (OHCB) - showed the best relative fit, although fit was marginal (CFI = 0.858). Despite pronounced floor effects, total-score reliability was acceptable to good (ordinal α and ωt = 0.81). The institutional sector was the most consistent independent determinant of global scores (*p* = 0.004). Some non-public sectors showed more favorable global scores than the public sector. The EL domain was the most sensitive to caregiver profiles showing exploratory associations with gender (*p* = 0.009), area of training (*p* = 0.043), and age-experience interaction (*p* = 0.007). Although men had more favorable beliefs, this requires caution as gender invariance was not established. Regarding SE, specific oral health training was the only significant predictor (*p* = 0.034). Item-level diagnostics identified items 3, 4, and 24 as weak.

**Conclusion:**

The NDCBS-PT demonstrated sufficient reliability and preliminary structural support for the four-factor model among Portuguese healthcare professionals, though requiring further refinement. Caregivers’ beliefs appear to be influenced by organizational settings and professional backgrounds. Oral-health training was linked to better self-efficacy and merits prospective testing.

## 1 Introduction

Oral health is a major indicator of systemic health and quality of life (1). Poor oral health has been linked to pain, malnutrition, systemic inflammation, cardiovascular disease, diabetic complications, and respiratory infections, including aspiration pneumonia, especially among older adults (2, 3). The worldwide increase in life expectancy has led to a substantial growth in the older adult population, accompanied by a higher prevalence of chronic diseases, multimorbidity, frailty, and functional dependence (4, 5).

Although oral health is widely recognized as an essential component of overall health, it is often overshadowed by other clinical tasks in nursing practice. A primary reason for the undervaluation of oral healthcare is the lack of awareness, knowledge, and training among healthcare professionals (6, 7). Oral health education and training are insufficient and rarely integrated into the nursing curriculum (7–9). Recent international reviews underscore this educational deficit, revealing that fewer than 30% of nursing programs include structured oral health teaching, and many graduates receive fewer than five hours of instruction on the topic throughout their degree program (7, 8). This limited exposure leaves many graduates unprepared to identify oral disorders or manage the complex needs of dependent patients. This problem is further complicated by critical barriers, which the literature categorizes into three groups: user-related (e.g., uncooperative behavior or resistance to care), organizational (e.g., lack of time, staff, and adequate materials), and caregiver-related (e.g., feelings of revulsion toward the procedure or fear of harming the patient) (6). The growing challenges posed by an aging population and the resulting increase in functional dependency have made oral care more important, evolving from a neglected task to a core clinical responsibility (7, 8, 10, 11).

In Portugal, structural inequalities in access to oral healthcare may exacerbate these challenges further. Dental care is primarily provided through the private sector and is only partially integrated into the National Health Service (NHS) (12, 13). Consequently, access to care is often determined by a patient’s socioeconomic status or institutional setting rather than clinical need. These systemic inequities place an uneven burden on caregivers, who must rely on their beliefs and self-efficacy to compensate for the lack of specialized professional support (14). Therefore, identifying the determinants of these beliefs is crucial for mapping organizational gaps.

The Nursing Dental Coping Belief Scale (NDCBS) was adapted and modified in 2005 (15) from the original Dental Coping Belief Scale (DCBS) - a 44-item instrument first tested among U. S. veterans (16). The original DCBS was designed to evaluate the impact of oral hygiene instructions on patient beliefs and health-related behaviors (10, 15). In contrast, the NDCBS focuses on clinical prioritization and supervision of care provided by healthcare staff. It is important to note that the term “nursing” in the scale name does not refer exclusively to licensed nurses. Rather, it refers generically to individuals who provide care in a nursing context, including medical assistants, formal caregivers, and health students, among others (15).

The NDCBS is a 28-item psychometric instrument with four equally weighted domains, each of which is represented by seven items: the Oral Healthcare Beliefs (OHCB) dimension assesses perceptions of preventive oral health behaviors; the External Locus of Control (EL) dimension assesses the conviction that oral health outcomes are determined by chance or external factors beyond one’s control; the Internal Locus of Control (IL) dimension reflects the conviction that one can influence oral health through personal actions; and finally, the Self-efficacy (SE) dimension evaluates confidence in one’s ability to perform oral care tasks effectively.

This study aimed to adapt and validate the NDCBS for the Portuguese healthcare professionals, resulting in the NDCBS-PT. Portugal’s unique healthcare landscape necessitated the adaptation of this scale. Oral health care is predominantly private and based on out-of-pocket payments, with only limited integration into the National Health Service (NHS). This structural exclusion creates significant health disparities, leaving care-dependent and vulnerable populations dependent on nurses and assistants for direct monitoring, making these professionals the de facto first line of oral health surveillance. However, these professionals often lack the structured training necessary to perform these tasks effectively.

This study examined the translation process, temporal stability, internal consistency, and structural validity to provide evidence of the NDCBS-PT’s suitability for exploring oral health beliefs and clinical gaps within this national context. The findings provide essential, evidence-based information for developing public health policies and targeted educational interventions that improve care provision and mitigate systemic risks, such as aspiration pneumonia, in dependent individuals. The multi-sectoral approach involving 304 professionals from primary care, hospitals, and social services allows for the identification of systemic and institutional determinants that influence oral healthcare priorities within the national workforce.

## 2 Methods

### 2.1 Design and participants

This cross-sectional, methodological study aimed to translate, culturally adapt, and validate the Portuguese version of the Nursing Dental Coping Belief Scale (NDCBS-PT). The translation process adhered to a rigorous, five-step international protocol to ensure semantic, idiomatic, and conceptual equivalence between the original English scale and the Portuguese version.

The study was conducted in accordance with the Declaration of Helsinki, as part of a research project approved by the Ethics Committee of Egas Moniz School of Health and Science (approval nos. 1,182 and 1,183, January 2023). The questionnaire was administered in a strictly anonymous format, to ensure that no personal identifiable information was collected. The anonymous and voluntary nature of the survey was explained in the introductory page, and participants could only proceed after indicating their agreement.

The target population was a diverse group of healthcare professionals, including registered nurses from various specialties, nurse and medical assistants, formal caregivers, health technicians, and allied health professionals, such as occupational therapists, speech therapists, physiotherapists, psychologists, and social workers. Participants were recruited from various clinical settings across Portugal, including hospitals, primary care units, and nursing homes. Eligibility criteria included being a native Portuguese speaker, being at least 18 years old, and being currently employed as a healthcare professional providing clinical care.

### 2.2 Procedures and data collection

Data collection was performed electronically using Microsoft Forms (Microsoft Corporation, Redmond, Washington, USA). The survey was distributed via a multi-channel strategy that included online links sent through institutional email lists and digital platforms. The data collection phase spanned 327 days, with an average completion time of nine minutes and 17 s per respondent.

As previously stated, the survey was completely anonymous, and participation was voluntary. Participants’ sociodemographic and professional characteristics were obtained using the same self-administered form used for the NDCBS-PT, solely to characterize the study sample. The collected sociodemographic variables included age, sex, academic qualifications, and work region (classified according to the 2024 NUTS II update). Professional characteristics were also retrieved, including area of training (profession), years of experience, primary clinical work settings (e.g., hospitals, primary care, or nursing homes), institutional sector (public/NHS, social/solidarity, private, or academic/other/mixed), target population served, and specific training in oral healthcare.

### 2.3 Cross-cultural adaptation of the Nursing Dental Coping Belief Scale

The NDCBS is a multidimensional instrument based on three behavioral psychology models: cognitive-behavioral, locus of control, and self-efficacy. The scale comprises 28 items organized into four conceptual domains, each with seven items (Figure 1). Responses were recorded on a 5-point Likert scale ranging from 1 (“Strongly disagree”) to 5 (“Strongly agree”). Before computing domain and total scores, item responses were harmonized where necessary so that lower item values consistently represented more favorable oral-health beliefs, stronger perceived control, and greater self-efficacy. Domain scores were calculated by summing the seven items within each domain and ranged from 7 to 35, while the total NDCBS-PT score ranged from 28 to 140. Therefore, lower final scores indicate more favorable beliefs.

**Figure 1:**
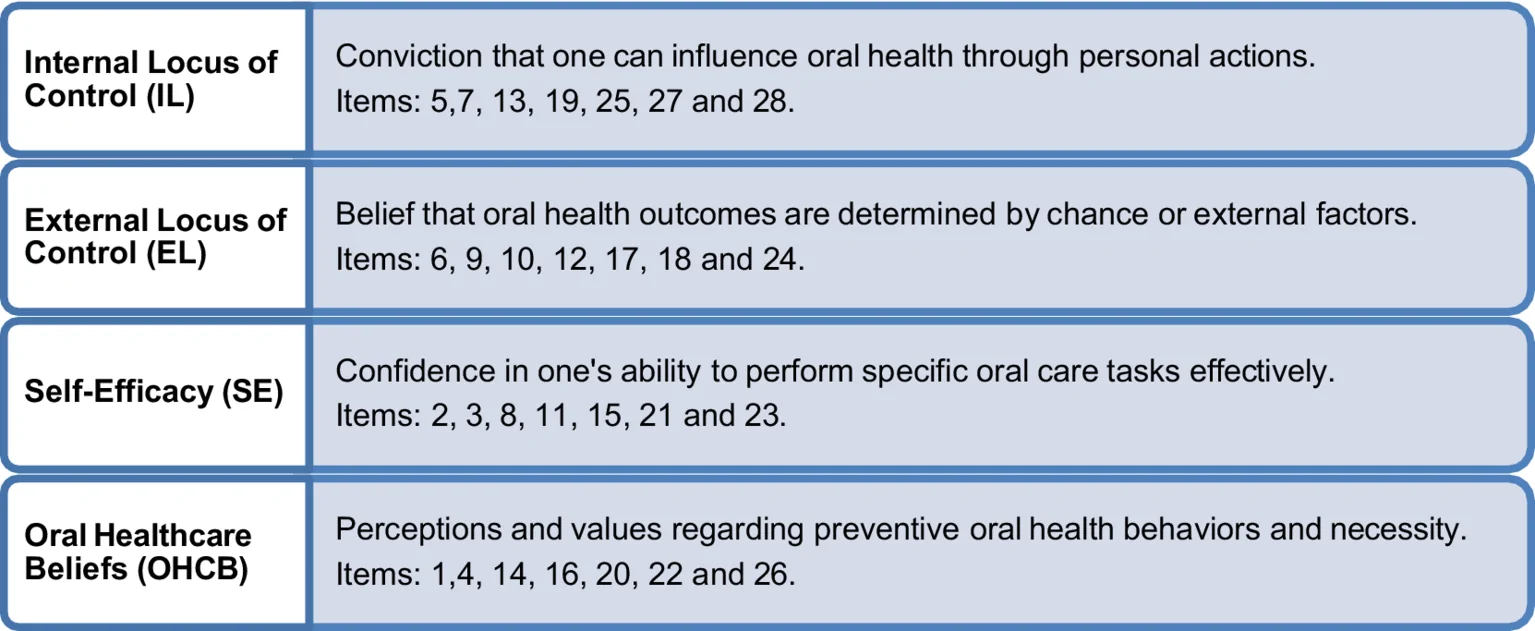
Conceptual mapping of the NDCBS 28 items within the four factor domains.

The adaptation process followed a five-stage protocol to ensure semantic, idiomatic, and conceptual equivalence (17, 18). First, two independent bilingual translators - a clinical expert and one layperson - translated the original scale from English to Portuguese. Then, two clinical experts compared and consolidated these versions into a single Portuguese version. Next, two independent native English speakers who were blinded to the original scale backtranslated this version into English. Finally, an expert committee composed of linguists and dental researchers reviewed all versions, resolving discrepancies to produce the pre-final version. Finally, a pilot test was conducted with 20 healthcare professionals to evaluate linguistic clarity and feasibility (Figure 2). Table 1 summarizes the outcome of this multistage adaptation process by providing a side-by-side comparison of the original 28 English items and their Portuguese translations.

**Figure 2:**
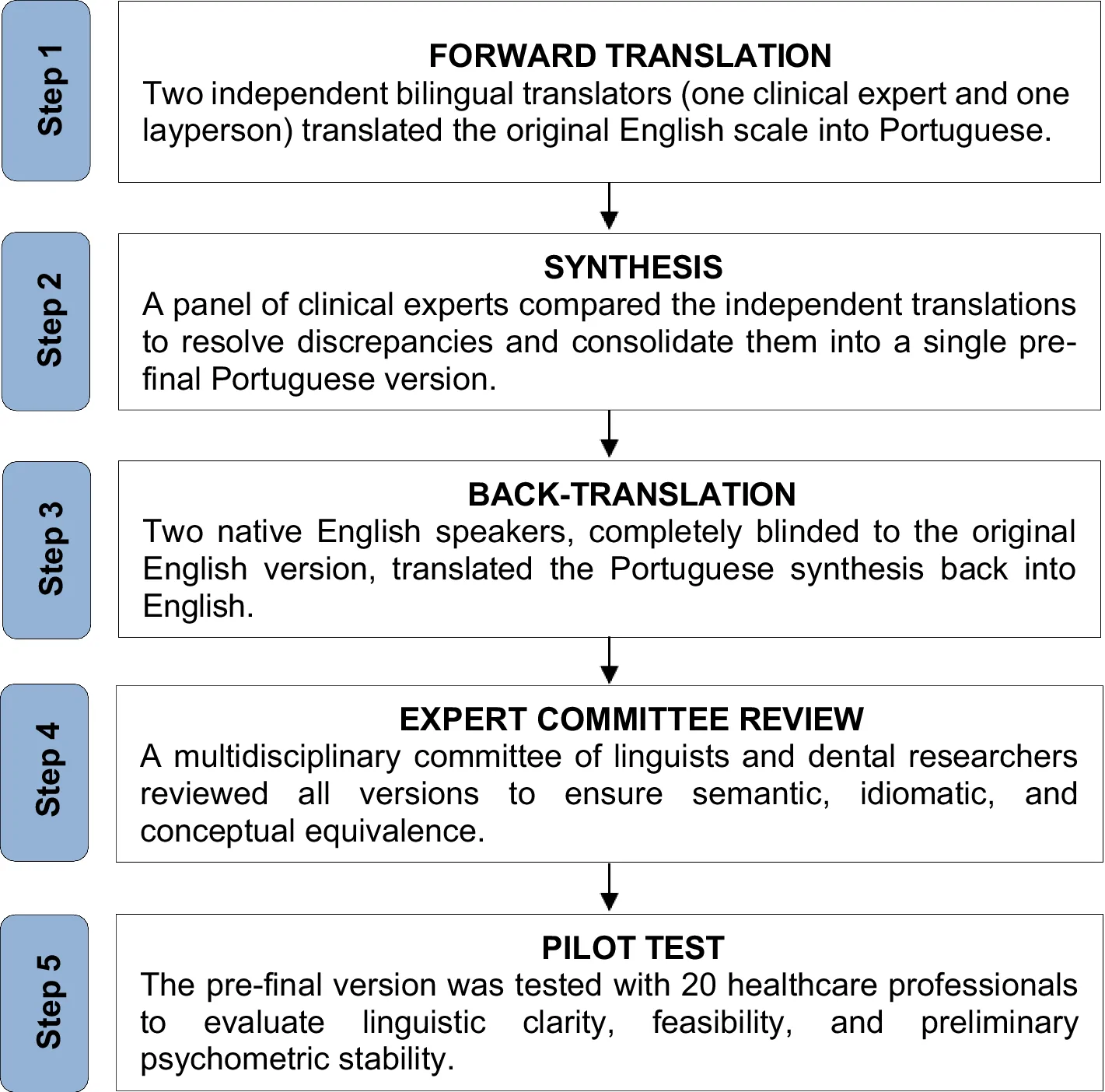
Flowchart of the five-step cross-cultural adaptation and translation protocol for the Portuguese version of the Nursing Dental Coping Belief Scale (NDCBS-PT). The process followed international guidelines adapted from Beaton et al. (18) and Herdman et al. (17).

**Table 1:** Original version and Portuguese translation of the Nursing Dental Coping Belief Scale (NDCBS-PT).

	Original item (English)	Portuguese translation (NDCBS-PT)	Domain
1	I believe that the patients themselves report when oral healthcare assistance is needed. ^R^	Acredito que os próprios pacientes informam quando é necessária assistência na sua higiene oral. ^R^	OHCB
2	If I brush and floss correctly, I expect fewer dental problems.	Se escovo os dentes e uso o fio dentário corretamente, é expectável que tenha menos problemas dentários.	SE
3	I believe that I know how different oral mucosal disorders can be treated.	Acredito que sei como diferentes doenças da mucosa oral podem ser tratadas.	SE
4	I believe that fluoride products are most suitable for children. ^R^	Acredito que os produtos fluoretados são mais indicados para crianças. ^R^	OHCB
5	I believe teeth should last a lifetime.	Acredito que os dentes devem durar uma vida inteira.	IL
6	Only the dentist can prevent cavities and gum disease.^R^	Apenas o Médico Dentista consegue prevenir a cárie dentária e as doenças gengivais.^R^	EL
7	I believe cavities can be prevented.	Acredito que a cárie dentária pode ser prevenida.	IL
8	If I were given oral healthcare training, I would be able to practice better oral healthcare.	Se eu recebesse formação sobre prestação de cuidados de saúde oral, seria capaz de prestar melhor estes cuidados.	SE
9	If both parents have had bad teeth, brushing and flossing will not help. ^R^	Se ambos os pais tiverem tido maus dentes, a escovagem e o uso do fio dentário não irão ajudar.^R^	EL
10	I believe that prostheses do not have to be removed during the night unless the patient wants to do so. ^R^	Acredito que as próteses dentárias não precisam de ser removidas durante a noite, a não ser que o paciente queira.^R^	EL
11	I believe I know how to floss correctly.	Acredito que sei utilizar corretamente o fio dentário.	SE
12	It is not possible to prevent sickness and medicines destroying teeth.^R^	É impossível prevenir que certas doenças e medicamentos prejudiquem os dentes.^R^	EL
13	I believe flossing teeth can help prevent gum disease.	Acredito que o uso do fio dentário pode ajudar a prevenir as doenças gengivais.	IL
14	Once gum disease has started, it is almost impossible to stop it. ^R^	Uma vez instalada a doença gengival, é quase impossível parar a sua progressão.^R^	OHCB
15	I believe I know how to prevent oral candidiasis.	Acredito que sei como prevenir a candidíase oral.	SE
16	If the gums bleed when you floss, this usually means that you should stop flossing. ^R^	Se as gengivas sangram quando se usa o fio dentário, significa que se deve parar a sua utilização.^R^	OHCB
17	I believe tooth loss is a normal part of growing old. ^R^	Acredito que a perda de dentes é uma consequência normal do processo de envelhecimento. ^R^	EL
18	Even if you take good care of your teeth, they are only going to fall out as you get older. ^R^	Mesmo que cuide bem dos seus dentes, eles irão cair à medida que envelhece.^R^	EL
19	I believe that our patients want me to offer help with oral care.	Acredito que os meus pacientes requerem a minha ajuda nos cuidados de saúde oral.	IL
20	I believe visiting the dentist is only necessary when experiencing pain. ^R^	Acredito que é necessário ir ao Médico Dentista apenas quando existe dor.^R^	OHCB
21	If I knew the facts about dental disease, I would be able to practice better oral care.	Se eu conhecesse os factos sobre as doenças orais, seria capaz de prestar melhores cuidados de saúde oral.	SE
22	I believe dentures are less trouble than taking care of natural teeth. ^R^	Acredito que a manutenção das próteses dentárias requer menos trabalho do que cuidar dos dentes naturais.^R^	OHCB
23	I believe I can successfully remove the majority of plaque to help prevent cavities and gum disease.	Acredito que sou capaz de remover, com sucesso, a maioria da placa bacteriana de forma a ajudar a prevenir a cárie dentária e a doença gengival.	SE
24	I believe that one method of brushing is just as effective as any other. ^R^	Acredito que qualquer técnica de escovagem dentária é eficaz.^R^	EL
25	I believe gum diseases can be prevented.	Acredito que as doenças gengivais podem ser prevenidas.	IL
26	If the gums bleed when you brush, this usually means that you should stop brushing. ^R^	Se as gengivas sangrarem durante a escovagem dentária, significa que devemos parar de escovar.^R^	OHCB
27	I believe that our patients eat better if they have a healthy, clean mouth.	Acredito que os nossos pacientes comem melhor se tiverem uma boca saudável e corretamente higienizada.	IL
28	I believe brushing can prevent cavities.	Acredito que a escovagem dos dentes pode prevenir a cárie dentária.	IL

^R^indicates reverse-worded items. All items were scored or recoded as necessary before calculating domain and total scores so that lower final scores consistently indicated more favorable oral-health beliefs. Responses were rated on a 5-point Likert scale: 1 = “Strongly disagree,” 2 = “Disagree,” 3 = “Neutral,” 4 = “Agree,” and 5 = “Strongly agree” (Portuguese: 1 = “Discordo totalmente”; 2 = “Discordo”; 3 = “Não concordo nem discordo”; 4 = “Concordo”; 5 = “Concordo totalmente”). The items are organized into four conceptual domains: Internal Locus of Control (IL), External Locus of Control (EL), Self-efficacy (SE), and Oral Health Care Beliefs (OHCB).

### 2.4 Sample size calculation

The sample size for this study was determined based on quality criteria for health status questionnaire measurement properties established by Terwee et al. (19). These guidelines recommend a ratio of 4 to 10 subjects per item and a minimum absolute sample size of 100 participants to ensure the stability of the variance–covariance matrix during factor analysis. Since the NDCBS-PT has 28 items, a minimum of 280 participants was needed to meet the “rule of thumb” of a 10:1 ratio and to provide sample size adequacy for confirmatory factor analysis (CFA). Our final validation sample of 304 healthcare professionals exceeds this requirement and surpasses the 7:1 ratio often cited as necessary for robust internal consistency and structural validity ratings (196 participants). It should be noted that the 10:1 ratio followed here is a general heuristic recommended for factor analysis stability rather than a model-specific assessment of statistical power or precision for ordinal indicators. Nevertheless, the sample size is statistically adequate to yield stable psychometric results for the Portuguese version of the scale.

A pilot study was conducted to evaluate the feasibility, linguistic clarity, and preliminary psychometric stability of the translated NDCBS-PT. Twenty healthcare professionals participated in a test–retest procedure with a one-week interval between administrations. This interval was chosen to minimize memory effects while ensuring that no significant changes in the participants’ underlying oral health beliefs occurred. The initial recruitment target was 10% of the validation sample (*n* = 28), but the final pilot size was limited to 20 individuals due to their high clinical workload and time constraints.

### 2.5 Statistical analyses

#### 2.5.1 Reliability

The reliability of the NDCBS-PT was evaluated through temporal stability and internal consistency. For the pilot study (*n* = 20), temporal stability was quantified by Intraclass Correlation Coefficients (ICC) for total and domain scores (two-way random-effects model) and by item-level stability via weighted Kappa coefficients with squared weights. Standard Error of Measurement (SEM) and the Minimal Detectable Change (MDC95) were calculated using the mean score metric for each domain and the total scale. To facilitate interpretation alongside the summed domain and total scores, raw-score equivalents were obtained by multiplying domain-level values by seven and total-scale values by 28. MDC95 was calculated as SEM × 1.96 × √2.

Internal consistency for the full validation sample (*n* = 304) was evaluated using Cronbach’s α, ordinal α (based on a polychoric correlation matrix), and omega total (ωt). Reliability inferences were primarily based on ordinal α and ωt rather than traditional Cronbach’s α. This is because the NDCBS scale uses five-category Likert indicators with skewed distributions, in which responses are heavily concentrated in favorable beliefs. In such situations, traditional Cronbach’s α tends to underestimate reliability.

#### 2.5.2 Descriptive analysis and construct validity

Descriptive statistics for each scale domain and total scores were reported as means, standard deviations (SD), and observed ranges. Item-level distributions were inspected to assess floor and ceiling effects, defining a threshold of 15% to identify significant response skewness.

Structural validity was examined through Confirmatory Factor Analysis (CFA) using the Weighted Least Squares Mean and Variance (WLSMV) estimator, which is robust for ordered-categorical indicators and non-normal distributions. We tested a hypothesized four-factor correlated model and compared its performance against competing structures, including single-factor, second-order, and bifactor models. Model fit was evaluated using the Comparative Fit Index (CFI), the Tucker-Lewis Index (TLI), the Root Mean Square Error of Approximation (RMSEA) with a 90% confidence interval (CI), and the Standardized Root Mean Square Residual (SRMR).

Convergent validity at the construct level was quantified using Composite Reliability (CR) and Average Variance Extracted (AVE). Associations and potential overlap between domains were examined using standardized latent factor correlations derived from the retained four-factor CFA model.

## 3 Results

### 3.1 Reliability

In the pilot study (*n* = 20), the NDCBS-PT demonstrated excellent data completeness (100%), with no missing values on any items. The total score showed good temporal stability over the one-week interval, with an intraclass correlation coefficient (ICC) of 0.774 (95% confidence interval [CI]: 0.509–0.904) (Table 2). At the domain level, the ICC values ranged from 0.616 for oral healthcare beliefs (OHCB) to 0.710 for self-efficacy (SE), indicating satisfactory reproducibility. Item-level stability, as measured by weighted kappa (κw), showed a median value of 0.403. Items 10 (“I believe that prostheses don’t have to be removed during the night unless the patient wants to do so”), 11 (“I believe I know how to floss correctly”), and 13 (“I believe flossing teeth can help prevent gum disease”) were the most stable, with κw of 0.742, 0.722, and 0.718, respectively (Supplementary Table S1). On the mean-item metric, the total SEM was 0.138, and the MDC95 was 0.382 (Table 2). These values correspond to approximately 3.86 and 10.70 points, respectively, on the raw 28–140 total-score scale. Therefore, changes in total NDCBS-PT scores should exceed approximately 11 raw-score points to be interpreted as exceeding measurement error at the 95% confidence level.

**Table 2:** Temporal stability and measurement error of the NDCBS-PT (pilot study, *n* = 20).

Scale/Domain	Cronbach’s α	ICC (95% CI)	SEM*	MDC95*
IL	0.552	0.667 (0.339–0.852)	0.229 (1.60)	0.634 (4.44)
EL	0.460	0.705 (0.388–0.872)	0.273 (1.91)	0.757 (5.30)
SE	0.554	0.710 (0.402–0.874)	0.240 (1.68)	0.666 (4.66)
OHCB	0.358	0.616 (0.241–0.829)	0.266 (1.86)	0.737 (5.15)
Total NDCBS-PT	0.676	0.774 (0.509–0.904)	0.138 (3.86)	0.382 (10.70)

IL, internal locus of control; EL, external locus of control; SE, self-efficacy; OHCB, oral healthcare beliefs; ICC, intraclass correlation coefficient; SEM, standard error of measurement; MDC95, minimal detectable change at the 95% confidence level. *SEM and MDC95 are reported on the mean-score metric, with raw-score equivalents shown in parentheses. Domain raw-score equivalents were obtained by multiplying by 7, and total-score equivalents by multiplying by 28.

For the full validation sample (*n* = 304), the internal consistency of the total NDCBS-PT score was strong, with an ordinal alpha (α) of 0.81 and a McDonald’s omega total (ωt) of 0.81. These indices, derived from a polychoric correlation matrix, were consistently higher than traditional Cronbach’s alpha (α = 0.69), which underestimated reliability due to pronounced floor effects (more than 50% of the responses were in the most favorable category) and the ordinal nature of the indicators. At the domain level (see Table 3), Internal Locus of Control (IL) exhibited the highest reliability (Ordinal α = 0.78; ωt = 0.79), followed by OHCB (Ordinal α = 0.70; ωt = 0.71) and External Locus of Control (EL) (Ordinal α = 0.66; ωt = 0.67). The SE domain exhibited the lowest internal consistency (Ordinal α = 0.58; ωt = 0.60).

**Table 3:** NDCBS-PT descriptive statistics and reliability/validity indices (*n* = 304).

Scale domain	Mean (SD)	Observed range	Cronbach’s α	Ordinal α	ωt	CR	AVE
IL	11.22 ± 3.25	7–30	0.63	0.78	0.79	0.81	0.39
EL	16.91 ± 4.61	7–35	0.59	0.66	0.67	0.71	0.30
SE	14.26 ± 3.32	7–25	0.49	0.58	0.60	0.63	0.22
OHCB	14.49 ± 4.14	7–35	0.59	0.70	0.71	0.72	0.29
Total NDCBS	56.89 ± 9.49	34–86	0.69	0.81	0.81	—	—

IL, internal locus of control; EL, external locus of control; SE, self-efficacy; OHCB, oral healthcare beliefs. All domain and total scores were computed after scoring harmonization; lower scores indicate more favorable beliefs.

As detailed in Supplementary Table S2, the distribution of responses was significantly skewed toward favorable beliefs, with most items exhibiting pronounced floor effects exceeding the 15% threshold. This concentration was most extreme for item 20 (“I believe visiting the dentist is only necessary when experiencing pain”) (83.9%), item 27 (“I believe that our patients eat better if they have a healthy, clean mouth”) (81.6%), and item 28 (“I believe brushing can prevent cavities”) (77.6%). These distributional properties were consistent with the item-total diagnostics and factor loadings (λ), which were generally moderate. However, items 4 (“I believe that fluoride products are most suitable for children”) and 24 (“I believe that one method of brushing is just as effective as any other”) were poor indicators.

Lower NDCBS scores indicate more favorable dental coping beliefs. The IL domain had the lowest mean score, 11.22 (±3.25). The SE and OHCB domains followed with mean scores of 14.26 (±3.32) and 14.49 (±4.14), respectively. The EL domain had the highest mean score, 16.91 (±4.61). The total NDCBS-PT score averaged 56.89 (±9.49) with an observed range of 34 to 86. This range is markedly skewed toward the favorable end (28–140). Descriptive statistics of the NDCBS-PT scores are presented in Table 3.

At the construct level, CR values ranged from 0.63 to 0.81, suggesting acceptable composite reliability for IL, EL, and OHCB, but weaker reliability for SE. However, AVE values were below the conventional 0.50 threshold across all domains, indicating limited convergent validity at the latent-construct level. These findings suggest that, although the total scale showed acceptable internal consistency, the domain-level constructs should be interpreted with caution.

### 3.2 Participant’s description

A total of 304 participants enrolled in the validation phase, with a mean age of 44.5 years (SD = 11.2; range = 18–70). Among participants who disclosed their gender, 82.8% were female (251/303), while one participant preferred not to disclose this information. The sample was highly educated, with 79.3% holding a higher education degree. Participants reported a mean of 18.2 years of professional experience (SD = 11.5). Most of the participants were registered nurses (*n* = 196) or nursing/medical assistants (*n* = 48). The sample also included formal caregivers, allied health professionals, and other healthcare workers across several clinical settings, including hospitals (*n* = 171), nursing homes (*n* = 80), home care services (*n* = 54), and primary care centers (*n* = 43). Regarding institutional affiliation, the participants were distributed across four main sectors, with the majority operating within the public National Health Service (38.8%) and the social/solidarity network (37.5%). In terms of specific oral health education, most of the sample (62.8%) relied on informal self-learning or clinical practice, whereas 30.6% had received formal training and 6.6% had completed postgraduate or specialized training. Geographically, the sample was evaluated according to the NUTS II (2024 update) classification (20) and reflected urban clusters in Greater Lisbon (50.7%) and the West and Tagus Valley (14.1%). Detailed sociodemographic data and descriptive statistics are provided in Table 4.

**Table 4:** Sociodemographic characteristics, professional background, and oral health training (*n* = 304).

Variable	Result
Sociodemographic data	
Age (years), Mean (SD) [min-max]	44.5 (11.2) [18–70]
Female (*n* = 251), Mean (SD) [min-max]	44.9 (11.1) [18–70]
Male (*n* = 52), Mean (SD) [min-max]	42.9 (11.7) [23–68]
Gender (Female), % (*n*)	82.8% (251)
Academic qualifications, % (*n*)	
Higher Education (Bac./Master/PhD)	79.3% (241)
Secondary Education	10.8% (33)
Professional/Technical Training	6.6% (20)
Basic Education	3.3% (10)
Professional background	
Profession, % (*n*)	
Registered Nurses (various specialties)	64.5% (196)
Nurse and Medical Assistants	15.8% (48)
Formal Caregivers	3.6% (11)
Allied Health Professionals*	5.6% (17)
Other professionals**	10.5% (32)
Years of practice, Mean (SD) [min – max]	18.2 (11.5) [<1–45]
Institutional sector***, % (*n*)	
Public/NHS	38.8% (118)
Social/Solidarity	37.5% (114)
Private	15.5% (47)
Academic/Other/Mixed	5.3% (16)
Specific oral health training, % (*n*)	
Informal self-learning / Clinical practice	62.8% (191)
Formal Higher/Professional Degree	30.6% (93)
Postgraduate/Specialized Training	6.6% (20)
Work region (NUTS II 2024), % (*n*)	
Greater Lisbon	50.7% (154)
West and Tagus Valley	14.1% (43)
North	10.2% (31)
Algarve	9.5% (29)
Setúbal Peninsula	8.6% (26)
Center	3.3% (10)
Alentejo	2.3% (7)
Autonomous Region of the Azores	1.3% (4)

SD, standard deviation; NHS, National Health Service. One participant preferred not to disclose gender, therefore, gender-specific subgroup counts sum to 303. *Allied Health Professionals include Psychologists (*n* = 6), Social Workers (*n* = 4), Physiotherapists (*n* = 3), Occupational Therapists (*n* = 3), and Speech Therapist (*n* = 1). **Other professionals include physicians and dental assistants. ***Percentages may not sum to 100% due to a small proportion of missing values for this specific variable, which was addressed using a complete-case analysis approach.

### 3.3 Construct validity

The structural validity of the NDCBS-PT was evaluated using confirmatory factor analysis (CFA) with the weighted least squares mean and variance (WLSMV) estimator, which is suited for ordered categorical indicators. The hypothesized four-factor correlated model, comprising IL, EL, SE, and OHCB domains, demonstrated a suboptimal absolute fit, despite better relative fit than alternative tested structures: CFI = 0.858, TLI = 0.844, RMSEA = 0.081 (90% CI: 0.075–0.087), and SRMR = 0.098. While these indices indicate some level of misfit, this pattern aligns with the markedly skewed distributions and pronounced floor effects observed in the sample, in which more than 50% of the responses fell into the most favorable category.

Furthermore, the four-factor correlated model significantly outperformed alternative structures, such as single-factor, second-order, and bifactor specifications (Table 5). Therefore, the four-factor correlated model was retained as the most theoretically coherent and comparatively best-fitting solution, but the absolute fit indices do not support an unequivocally strong structural solution. Standardized factor loadings were generally moderate, ranging from 0.21 to 0.85 (see Supplementary Table S2). Specifically, item 18 (“Even if you take good care of your teeth, they are only going to fall out as you get older”) emerged as an excellent indicator of EL (λ = 0.85). Conversely, items 4 (“I believe that fluoride products are most suitable for children”) and 24 (“I believe that one method of brushing is just as effective as any other”) were identified as weak indicators (λ = 0.21 and 0.26, respectively) and had the lowest corrected item-total correlations (r < 0.10). Furthermore, item 3 (“I believe that I know how different oral mucosal disorders can be treated”) stood out as an anachronism, being the only indicator with a negative item-total correlation (r = −0.074) regarding the total scale score.

**Table 5:** Model fit indices for the hypothesized NDCBS-PT structure and alternative models (*n* = 304).

Model specification	χ2	df	CFI	TLI	RMSEA (90% CI)	SRMR	ΔCFI
4-factor correlated	1027.2	344	0.858	0.844	0.081 (0.075–0.087)	0.098	—
Bifactor model	1085.7	322	0.816	0.794	0.095 (0.089–0.101)	0.094	−0.042
2nd-order model	1140.9	346	0.794	0.779	0.097 (0.091–0.103)	0.104	−0.064
1-factor model	1824.5	350	0.691	0.666	0.118 (0.112–0.123)	0.126	−0.166

CFI, Comparative Fit Index; TLI, Tucker-Lewis Index; RMSEA, root mean square error of approximation; SRMR, standardized root mean square residual. ΔCFI is reported relative to the hypothesized four-factor model.

### 3.4 Gender invariance and latent relationships

A multigroup CFA was conducted to evaluate the invariance of the NDCBS-PT across gender. We estimated four successive models with increasing levels of constraint: (1) unconstrained; (2) factor loadings constrained (Model 1); (3) factor loadings and structural covariances constrained (Model 2); and (4) factor loadings, structural covariances, and measurement residuals constrained (Model 3). Formal measurement invariance across gender groups was not established. Therefore, the four-factor correlated model was retained as the most theoretically coherent and comparatively best-fitting solution, but the absolute fit indices do not support an unequivocally strong structural solution.

Standardized latent factor correlations derived from the retained four-factor CFA model were used to examine the relationships between the four NDCBS-PT domains (Table 6). The strongest associations were observed between OHCB and EL (r = 0.738) and between SE and IL (r = 0.729), suggesting partial conceptual overlap between these domain pairs. The remaining correlations were weak to moderate, supporting some empirical separability among domains, although the stronger correlations indicate that discriminant validity is not unequivocal for all constructs.

**Table 6:** Latent factor correlations between NDCBS-PT domains (*n* = 304).

NDCBS-PT domains	OHCB	SE	EL	IL
Oral healthcare beliefs (OHCB)	1.000	—	—	—
Self-efficacy (SE)	0.172	1.000	—	—
External locus of control (EL)	0.738	0.078	1.000	—
Internal locus of control (IL)	0.515	0.729	0.193	1.000

Values are standardized latent factor correlations derived from the retained four-factor correlated CFA model. The two strongest correlations, OHCB–EL and SE–IL, are highlighted because they were interpreted as evidence of partial construct overlap.

### 3.5 Sociodemographic and professional factors associated with NDCBS-PT scores

In addition to cross-culturally validating the Portuguese translation of the NDCBS, collecting sociodemographic and professional data enabled an analysis of oral health beliefs among the Portuguese healthcare professionals. This analysis permitted a comparison of these beliefs against individual and institutional characteristics. The multivariable association analysis was conducted using a subset of participants (n = 268), due to the adoption of a complete-case analysis approach. While the 28 NDCBS-PT items exhibited 100% data completeness with no missing values, certain sociodemographic and professional covariates had missing data or non-harmonizable responses. To ensure stability and comparability of regression coefficients across all adjusted models, participants with incomplete information for any predictor variable were excluded from these analyses.

The fully adjusted multivariable analysis suggested that NDCBS-PT scores were more consistently associated with institutional context than with individual characteristics alone (Table 7). The institutional sector (public/NHS, social/solidarity, private, or academic/other/mixed.) was the most consistent independent determinant, showing significant associations with OHCB (*p* = 0.012), EL (*p* = 0.027), and the global NDCBS-PT score (*p* = 0.004). Specifically, professionals working in the social and academic sectors exhibited global beliefs that were significantly more favorable than those working in the public sector (NHS).

**Table 7:** Multivariable associations between participant characteristics and NDCBS-PT scores (*n* = 268).

Predictor variables	OHCB (p)	SE (p)	EL (p)	IL (p)	Global score (p)
Sex (Male vs. Female)	0.885	0.901	**−1.55 (0.009)**	0.541	0.170
Age (z-score)	0.387	0.745	0.478	0.838	0.410
Years of practice (z-score)	0.721	0.878	0.576	0.965	0.578
Educational level	0.384	0.507	0.124	0.752	0.332
Area of training	0.121	0.871	−3.24 (0.043)*	0.864	−5.54 (0.049)*
Specific oral-health training	0.970	**0.034**	0.276	0.211	0.067
Institutional sector	**0.012**	0.468	−2.64 (0.027)*	0.516	−5.62 (0.004)*
Target population	0.777	0.338	0.183	0.972	0.512
Age × Experience interaction**	—	—	**0.66 (0.007)**	**—**	—

OHCB, Oral Health Care Beliefs; SE, Self-efficacy; EL, External Locus of Control; IL, Internal Locus of Control. Values are omnibus *p*-values derived from fully adjusted robust linear models using HC3 standard errors and Type II ANOVA. They indicate evidence of association between each predictor and each NDCBS-PT score. Bold values indicate *p* < 0.05. For clarity, statistically significant coefficients (B) are reported with their respective *p*-values. Non-significant associations only report the omnibus *p*-value. *For categorical variables with more than two levels, the reported B reflects the most significant contrast (e.g., academic vs. public for sector; nursing vs. non-health for area of training). The *p*-value represents the omnibus test for the entire variable. **The interaction term was estimated only for the EL domain (B = 0.66, *p* = 0.007), indicating that the impact of experience on external beliefs is moderated by life stage.

The EL domain was the most sensitive dimension to caregiver profiles, yielding several significant associations (Table 7). In the adjusted exploratory analysis, sex was associated with EL scores (B = −1.55; *p* = 0.009). Although men had lower EL scores than women, suggesting more favorable external locus beliefs, this result should not be interpreted as definitive evidence of gender differences, as formal measurement invariance across gender groups was not established. Furthermore, the area of training influenced both EL (*p* = 0.043) and the global score (*p* = 0.049). Nursing and advanced health training groups demonstrated more robust perceptions of control over external factors (EL) than groups from non-health backgrounds. A significant age-experience interaction was detected within this domain (*p* = 0.007), suggesting that the impact of professional practice years on beliefs about external determinants (e.g., luck or fate) varies by the professional’s stage of life. Regarding SE, the only statistically significant factor was specific oral health training (*p* = 0.034), indicating that targeted instruction enhances confidence in performing oral care.

Conversely, variables such as age, years of practice (when analyzed independently), educational level, and target population served did not show independent associations with any domain (all *p* > 0.05, see Table 7).

## 4 Discussion

The psychometric evaluation of the NDCBS-PT provides qualified support for its use in assessing oral health beliefs among Portuguese healthcare professionals.

Using the weighted least squares mean and variance adjusted (WLSMV) estimator, which is specifically suited to ordinal-categorical data, confirmatory factor analysis (CFA) demonstrated that this multidimensional model significantly outperforms alternatives. The model successfully maintains the hypothesized four-factor, correlated structure originally established in the Swedish (15) development study: Internal Locus of Control (IL), External Locus of Control (EL), Self-Efficacy (SE), and Oral Healthcare Beliefs (OHCB). The total score exceeded the commonly recommended thresholds for acceptable internal consistency in psychometric instruments (ordinal α = 0.81; ωt = 0.81), supporting the scale’s reliability. Although the domain-level indices were modest, this was largely due to pronounced floor effects, with over 50% of the responses concentrated in the most favorable category. These findings do not invalidate the scale, but they qualify the strength of the validity evidence and suggest that interpretation should be more cautious at the domain and item levels than at the total-score level.

There was mixed evidence for construct-level validity. Convergent validity was limited as the average variance extracted (AVE) values fell below 0.50 across all domains. The high latent correlations between OHCB-EL (r = 0.74) and SE-IL (r = 0.73) suggest partial construct overlap and indicate that these belief domains are closely related among Portuguese caregivers and are consistent with the scale’s theoretical framework. The strong association between SE-IL reflects the shared conceptual roots of personal agency. While SE assesses confidence in performing specific oral care tasks, IL represents the broader conviction that personal actions can effectively influence patient outcomes. Similarly, the OHCB-EL correlation highlights how preventive beliefs in this population are often tied to the perceived influence of external factors, such as the dentist’s role or the inevitable impact of aging. These factors are core components of the external locus of control.

Furthermore, item-level diagnostics identified Items 3, 4, and 24 as weak indicators. The negative correlation (r = −0.074) found for Item 3 (“I believe that I know how to treat different oral mucosal disorders”) is particularly noteworthy. It may reflect semantic ambiguity or cultural differences in how Portuguese caregivers perceive their roles in clinical diagnosis versus daily hygiene monitoring.

The total NDCBS-PT mean score (56.89 ± 9.49) indicates that Portuguese healthcare professionals generally have favorable beliefs about oral health. The IL domain was the most robust (11.22 ± 3.25), reflecting professionals’ strong perception of themselves as primary agents capable of influencing patient outcomes. This finding aligns with recent Norwegian research, which revealed a similar preventive orientation among home care personnel (9). However, international evidence suggests a potential self-assessment bias: although nurses often report optimistic beliefs, their actual clinical practice may be hindered by inadequate training during their undergraduate education (6).

While previous studies in Sweden (10) and Finland (21) emphasized the workplace setting (e.g., home care versus nursing homes), in the Portuguese context, the institutional sector emerged as the factor most consistently associated with oral health belief scores (*p* = 0.004), rather than individual experience or age, primarily determines oral health beliefs. Professionals in the academic (B = −5.62) and social (B = −3.32) sectors had significantly more favorable beliefs than those in the public National Health Service (NHS). Interestingly, no differences were found between the public and private sectors. These findings suggest that disparities may stem from unique organizational cultures, such as regular access to continuing education and specialized protocols in academic and social networks (IPSS), rather than a simple public-private financial divide.

The EL domain was most sensitive to professional profiles. Men reported more favorable beliefs than women (B = −1.55, *p* = 0.009), which diverges from the literature in Sweden (10) and Norway (9). However, this association is preliminary due to the predominance of females in the sample (82.6%) and the lack of established gender invariance. This discrepancy may reflect the particular occupational distribution of the Portuguese workforce rather than a consistent gender disparity. Notably, we detected a significant age-experience interaction in the EL domain (*p* = 0.007), indicating that the impact of practice years is moderated by life stage. This likely reflects the “fading effect” of initial education among senior professionals who have practiced for decades in a system with historically limited public dental coverage.

Finally, specific oral health training was the sole predictor of self-efficacy (*p* = 0.034), reflecting patterns observed in Finland (21) and Norway (9). This finding suggests that, although professionals value prevention, clinical confidence requires targeted instruction. The “transversal” consistency of these beliefs across pediatric and geriatric settings validates the NDCBS-PT as a stable and versatile diagnostic tool for mapping training needs in various Portuguese healthcare settings.

Driven by an aging population and the resulting increase in functional dependency (7, 8), as well as the link between poor oral hygiene and systemic conditions (especially aspiration pneumonia) in geriatric and critical care settings (2, 8), oral care has transitioned from an overlooked task to a fundamental clinical responsibility. In Portugal, where public dental coverage is limited and costs are largely out-of-pocket, caregivers’ beliefs are the primary line of defense for vulnerable individuals who are dependent on others. Our sample’s high EL score (16.91 ± 4.61) is particularly revealing, suggesting that misconceptions, such as the belief that only dentists can prevent oral disease, persist among professionals. The NDCBS-PT is a vital diagnostic tool that maps these knowledge gaps. It enables health authorities to design targeted interprofessional training that empowers caregivers to mitigate oral disease in care-dependent settings.

### 4.1 Strengths and limitations

Although the validation was successful overall, several limitations remain. First, the sample was predominantly female (82.6%), reflecting the reality that women are overwhelmingly represented in nursing and formal caregiving professions. While this gender imbalance was anticipated, it limited our ability to establish formal measurement invariance across gender groups. Notably, studies in Sweden and Norway have found that male staff tend to have lower oral healthcare beliefs or a higher EL than their female counterparts (9, 10). Consequently, the observed association between sex and EL scores should be regarded as exploratory and should not be interpreted as definitive evidence of gender-based differences in oral health beliefs. Second, the pilot study failed to reach the ideal retest sample size of 10% (*n* = 28) because securing second responses within one week was impossible, likely due to the high workload and time constraints faced by health professionals. Third, recruitment was concentrated geographically in Greater Lisbon (50.7%) and the West and Tagus Valley (14.1%). While Portugal’s linguistic and cultural homogeneity suggests these findings may be generalized, future research should aim for broader national representation and longitudinal designs to assess the scale’s sensitivity to educational interventions. Finally, pronounced floor effects were observed, with over 50% of responses concentrated in the most favorable categories. This not only penalized the global fit indices, such as CFI (0.858) and TLI (0.844), but they may also limit the instrument’s ability to distinguish between professionals with generally favorable oral health beliefs. Discriminant validity diagnostics also revealed significant overlap between OHCB-EL and SE-IL. These findings provide a concrete roadmap for future linguistic refinement to reduce construct conflation.

## 5 Conclusion

The NDCBS-PT is a promising instrument for evaluating the oral health beliefs of a diverse group of Portuguese healthcare professionals in various care settings. Although it demonstrated adequate reliability and preliminary structural support for the four-factor model, it may benefit from future refinements to mitigate floor effects and construct overlap. Institutional sector was the main factor associated with overall belief scores, while external locus of control was found to be more sensitive to career path and life stage. Specific training was associated with greater self-efficacy, which confirms the instrument’s potential role in identifying training gaps. By identifying institutional settings and targeted training as key factors associated with these beliefs, the study offers preliminary, actionable insights into public health policy. Although structural support for the model is still in the early stages, improving the oral health literacy of the non-dental workforce is a cost-effective way to mitigate systemic health risks and promote health equity among Portugal’s most vulnerable populations.

## Data Availability

The raw data supporting the conclusions of this article will be made available by the authors, without undue reservation.
