# Supplementary material for: Nursing Dental Coping Belief Scale among Portuguese healthcare professionals (NDCBS-PT): validation and associated factors
Source: Front Public Health. 2026 Sep 21;14:1950334. doi: 10.3389/fpubh.2026.1950334 (PMC13635153; doi:10.3389/fpubh.2026.1950334)
Supplement: Supplementary file 1 [file Table_1.docx]

**Supplementary Table S1**. Item-weighted Kappa values (Pilot Study, n=20).

| Item | Domain | Kappa weighted (κw) | CI 95% |
| --- | --- | --- | --- |
| 1 | OHCB | 0.200 | -0.333 to 0.595 |
| 2 | SE | 0.399 | 0.181 to 0.815 |
| 3 | SE | 0.615 | 0.265 to 0.809 |
| 4 | OHCB | 0.615 | 0.184 to 0.801 |
| 5 | IL | 0.603 | 0.226 to 0.803 |
| 6 | EL | 0.203 | -0.087 to 0.611 |
| 7 | IL | 0.300 | -0.100 to 0.688 |
| 8 | SE | 0.407 | 0.000 to 0.815 |
| 9 | EL | 0.638 | -0.030 to 0.876 |
| 10 | EL | 0.742 | 0.417 to 0.904 |
| 11 | SE | 0.722 | 0.467 to 1.000 |
| 12 | EL | 0.271 | -0.132 to 0.584 |
| 13 | IL | 0.718 | 0.464 to 0.880 |
| 14 | OHCB | 0.345 | -0.132 to 0.671 |
| 15 | SE | 0.327 | -0.000 to 0.574 |
| 16 | OHCB | 0.567 | 0.138 to 0.829 |
| 17 | EL | 0.549 | 0.155 to 0.776 |
| 18 | EL | 0.587 | 0.241 to 0.813 |
| 19 | IL | 0.162 | -0.214 to 0.568 |
| 20 | OHCB | -0.053 | -0.136 to 0.000 |
| 21 | SE | 0.697 | 0.416 to 0.894 |
| 22 | OHCB | 0.123 | -0.385 to 0.620 |
| 23 | SE | 0.078 | -0.250 to 0.829 |
| 24 | EL | 0.594 | 0.167 to 0.883 |
| 25 | IL | 0.200 | -0.116 to 0.645 |
| 26 | OHCB | 0.615 | 0.362 to 0.833 |
| 27 | IL | 0.059 | -0.216 to 0.643 |
| 28 | IL | 0.062 | -0.286 to 0.571 |
| OHCB: Oral Healthcare Beliefs; SE: Self-efficacy; EL: External locus of control; IL: Internal locus of control; CI: Confidence Interval.  Values were derived from a test-retest procedure involving 20 healthcare professionals with a one-week interval. Stability was estimated using weighted Cohen’s kappa with squared weights, which is appropriate for the ordinal nature of the 1–5 Likert scale. Due to the small pilot sample size, 95% confidence intervals (CIs) were estimated via nonparametric bootstrap resampling with 2,000 replications. Item scoring was maintained in its original direction, where lower values represent more favorable or positive oral health beliefs. Median item-level stability was 0.403. Negative or near-zero values (e.g., item 20) were interpreted as preliminary diagnostic indicators of potential instability or semantic ambiguity that should be monitored during the full validation phase. | | | |
