# Supplementary material for: Nursing Dental Coping Belief Scale among Portuguese healthcare professionals (NDCBS-PT): validation and associated factors
Source: Front Public Health. 2026 Sep 21;14:1950334. doi: 10.3389/fpubh.2026.1950334 (PMC13635153; doi:10.3389/fpubh.2026.1950334)
Supplement: Supplementary file 2 [file Table_2.docx]

**Supplementary Table S2.** Distributional properties; CFA standardized factor loadings; floor/ceiling effects; Item-total correlations and alpha coefficients if the item is deleted (n=304).

| Item | Domain | Mean ± SD | Loading (λ) | Floor effect (%) | Ceiling effect (%) | Adjusted Item-Total Correlation | Cronbach's alpha if an item is deleted |
| --- | --- | --- | --- | --- | --- | --- | --- |
| 1 | OHCB | 2.54 ± 1.24 | 0.33 | 21.1% | 6.6% | 0.169 | 0.685 |
| 4 | OHCB | 3.12 ± 1.26 | 0.21 | 14.5% | 13.8% | 0.098 | 0.692 |
| 14 | OHCB | 2.14 ± 1.05 | 0.59 | 31.9% | 2.3% | 0.417 | 0.664 |
| 16 | OHCB | 1.77 ± 1.00 | 0.72 | 51.0% | 3.0% | **0.474**** | 0.660 |
| 20 | OHCB | 1.38 ± 1.02 | 0.60 | 83.9% | 5.3% | 0.285 | 0.675 |
| 22 | OHCB | 1.88 ± 1.17 | 0.43 | 53.0% | 4.0% | 0.278 | 0.675 |
| 26 | OHCB | 1.66 ± 0.94 | 0.68 | 56.3% | 1.6% | 0.414 | 0.666 |
| 2 | SE | 1.46 ± 0.68 | 0.41 | 60.9% | 0.7% | 0.135 | 0.686 |
| 3 | SE | 2.71 ± 1.18 | 0.27 | 14.5% | 8.2% | **-0.074*** | 0.706 |
| 8 | SE | 1.57 ± 0.89 | 0.67 | 60.9% | 2.0% | 0.196 | 0.682 |
| 11 | SE | 1.80 ± 0.86 | 0.42 | 39.8% | 1.0% | 0.179 | 0.683 |
| 15 | SE | 2.36 ± 0.95 | 0.28 | 15.1% | 3.6% | 0.121 | 0.687 |
| 21 | SE | 1.63 ± 0.83 | 0.67 | 53.0% | 1.0% | 0.208 | 0.681 |
| 23 | SE | 2.73 ± 1.19 | 0.36 | 11.5% | 9.5% | 0.070 | 0.694 |
| 6 | EL | 1.79 ± 1.15 | 0.40 | 55.3% | 4.9% | 0.212 | 0.681 |
| 9 | EL | 1.83 ± 1.17 | 0.63 | 55.6% | 4.3% | 0.376 | 0.666 |
| 10 | EL | 2.07 ± 1.31 | 0.30 | 50.7% | 7.2% | 0.154 | 0.687 |
| 12 | EL | 3.44 ± 1.29 | 0.30 | 9.5% | 22.7% | 0.223 | 0.680 |
| 17 | EL | 2.35 ± 1.19 | 0.74 | 28.6% | 2.0% | 0.349 | 0.669 |
| 18 | EL | 2.45 ± 1.19 | 0.85 | 23.7% | 4.6% | 0.456 | 0.658 |
| 24 | EL | 2.98 ± 1.27 | 0.26 | 13.8% | 12.2% | 0.096 | 0.692 |
| 5 | IL | 2.34 ± 1.27 | 0.37 | 28.0% | 8.9% | 0.122 | 0.690 |
| 7 | IL | 1.38 ± 0.72 | 0.47 | 71.1% | 1.3% | 0.173 | 0.683 |
| 13 | IL | 1.71 ± 0.86 | 0.54 | 48.4% | 1.0% | 0.304 | 0.675 |
| 19 | IL | 1.71 ± 0.83 | 0.46 | 47.7% | 1.0% | 0.177 | 0.683 |
| 25 | IL | 1.54 ± 0.65 | 0.80 | 53.3% | 0.0% | 0.404 | 0.672 |
| 27 | IL | 1.27 ± 0.69 | 0.78 | 81.6% | 1.3% | 0.275 | 0.678 |
| 28 | IL | 1.28 ± 0.60 | 0.78 | 77.6% | 0.7% | 0.315 | 0.677 |
| OHCB: Oral Healthcare Beliefs, SE: Self-efficacy, EL: External Locus of Control, IL: Internal Locus of Control, CFA: Confirmatory Factor Analysis, SD: Standard Deviation.  All item scores were harmonized before analysis. Lower mean values represent more favorable oral health beliefs, stronger perceived control, and higher self-efficacy. Standardized loadings were derived from the hypothesized four-factor correlated model using the WLSMV estimator, which is suited for ordered categorical data. Percentages represent the proportion of respondents with the lowest (1) and highest (5) possible scores. Effects are considered significant if they exceed the 15% threshold.  * This item is the only indicator with a negative corrected item-total correlation (r = −0.074) and removing it would increase the total scale's Cronbach's alpha (from 0.69 to 0.71). This suggests semantic ambiguity or misinterpretation of its polarity.  ** Highlighted as having the strongest corrected item-total correlation within the OHCB domain. | | | | | | | |
